# Surgical training model and safe implementation of robotic pancreatoduodenectomy in Japan: a technical note

**DOI:** 10.1186/s12957-021-02167-9

**Published:** 2021-02-19

**Authors:** Kosei Takagi, Yuzo Umeda, Ryuichi Yoshida, Takahito Yagi, Toshiyoshi Fujiwara, Amer H. Zureikat, Melissa E. Hogg, Bas Groot Koerkamp

**Affiliations:** 1 Department of Gastroenterological Surgery, Okayama University Graduate School of Medicine, Dentistry, and Pharmaceutical Sciences, 2-5-1 Shikata-cho, Kita-ku, Okayama, 700-8558 Japan; 2 Department of Surgery, Erasmus MC, University Medical Center Rotterdam, Rotterdam, The Netherlands; 3 Division of Surgical Oncology, University of Pittsburgh Medical Center, Pittsburgh, PA USA; 4 Department of Surgery, North Shore Hospital, Chicago, IL USA

**Keywords:** Pancreatoduodenectomy, Robotic surgery, Training

## Abstract

**Background:**

Growing evidence for the advantages of robotic pancreatoduodenectomy (RPD) has been demonstrated internationally. However, there has been no structured training program for RPD in Japan. Herein, we present the surgical training model of RPD and a standardized protocol for surgical technique.

**Methods:**

The surgical training model and surgical technique were standardized in order to implement RPD safely, based on the Dutch training system collaborated with the University of Pittsburgh Medical Center.

**Results:**

The surgical training model included various trainings such as basic robotic training, simulation training, biotissue training, and a surgical video review. Furthermore, a standardized protocol on the surgical technique was established to understand the tips, tricks, and pitfalls of RPD.

**Conclusions:**

Safe implementation of RPD can be achieved through the completion of a structured training program and learning surgical technique. A nationwide structured training system should be developed to implement the program safely in Japan.

**Supplementary Information:**

The online version contains supplementary material available at 10.1186/s12957-021-02167-9.

## Background

The evidence of robotic pancreatoduodenectomy (RPD) has been growing internationally in the past decade [1]. However, RPD presents unique difficulties requiring advanced skills in both pancreatic and robotic surgery. Furthermore, there are still ongoing concerns regarding robotic approaches that will help create meaningful benefits during the learning curve or may lead to unacceptable postoperative outcomes [2]. A lack of convincing and high-quality data, including these issues, might limit the adoption and expansion of RPD. In Japan, the introduction and dissemination of RPD have been relatively late compared to those in the West. In these circumstances, we should learn from the lessons learned and pitfalls encountered with respect to the training model, surgical technique, and safe implementation of RPD from high-volume pancreatic centers in the world.

In The Netherlands, the Dutch Pancreatic Cancer Group initiated a multicenter nationwide training program on laparoscopic distal pancreatectomy (LAELAPS) [3] and laparoscopic PD (LAELAPS-2) [4]. Subsequently, the multicenter training program for RPD (LAELAPS-3) was established in collaboration with the University of Pittsburgh Medical Center. The LAELAPS-3 consists of simulation training, suturing training on artificial tissue, video training, and proctoring of the first procedures [5].

Herein, we develop a surgical training model for RPD in Japan, including a training system, standardization of surgical techniques, and safe implementation, based on experiences from the clinical fellowship in the Netherlands.

## Methods

### Prior to starting the program

#### Multidisciplinary team

The establishing of a dedicated multidisciplinary team should be of prime importance in the setup. The multidisciplinary team consists of experienced hepato-pancreato-biliary (HPB) surgeons, anesthesiologists, scrub nurses, ward nurses, physical therapists, and medical engineers. Not only surgeons but also anesthesiologists and scrub nurses should be dedicated to HPB surgery with extensive experience to ensure prompt standardization of the procedure. In addition, dedicated individual and team training should be implemented.

#### Patient selection

Although no contraindications based on patient’s age, obesity, or previous abdominal surgery have been suggested in the Miami guidelines [1], a body mass index between 20 and 35 kg/m^2^ is recommended in patients undergoing robotic pancreatic surgery [5]. The initial indication should be in selected patients with benign and low-grade malignant tumors. Patients with chronic pancreatitis and bulky tumors who might require vascular reconstruction should be excluded at the beginning [5].

#### Institution

Since center volume strongly affects postoperative outcomes following minimally invasive pancreatic surgery, RPD should be performed in high-volume pancreatic centers [1]. In Japan, only centers performing more than 50 pancreatic resections in a year are allowed to perform RPD. The prospective registry in the National Clinical Database (http://www.ncd.or.jp/) is mandatory in Japan.

#### Surgical training model

**Fig. 1:**
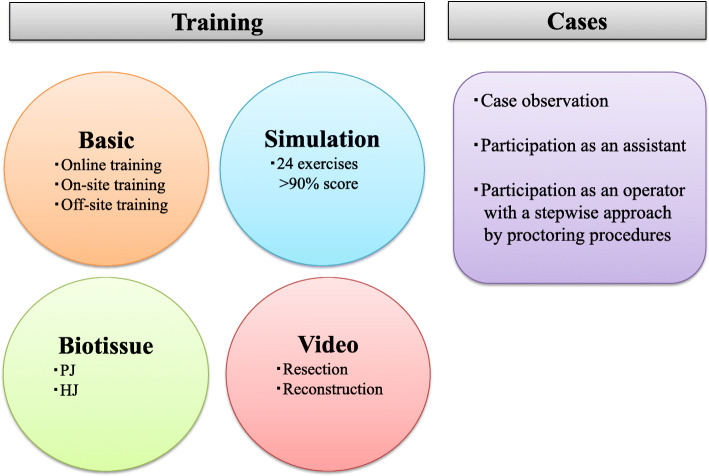
Overview of the structured training system for robotic pancreatoduodenectomy. PJ, pancreaticojejunostomy; HJ, hepaticojejunostomy

Surgeons involved in the program should have extensive experience in HPB surgery, as well as basic knowledge of the robotic system. Moreover, pancreatoduodenectomy remains a highly complex procedure requiring advanced suturing skills for anastomoses, including pancreaticojejunostomy (PJ) and hepaticojejunostomy (HJ). It is determined that immature anastomoses would increase the risk of postoperative complications, such as pancreatic fistula and biliary complications. Therefore, specific training should be essential before performing the first procedure. We have developed a structured training model consisting of basic robotic training, simulation training, video conference, and biotissue training, as illustrated in Fig. 1.

#### Basic robotic training

Prior to the commencement of robotic surgery, trainees are required to complete the da Vinci System Training (Intuitive Surgical, CA, USA) in order to get accustomed to the fundamental use of the da Vinci surgical system. The training pathway consists of online training, on-site and off-site training, and case observations.

#### Simulation training

Surgical simulation has been developed as a valid tool for the training and assessment of objective surgical skills [6]. In addition, the advantages of the virtual reality robotic simulation curriculum have been demonstrated [7]. In the training simulation system of the da Vinci surgical system, two simulation platforms with 24 virtual reality exercises (the Intuitive Surgical Backpack Simulator and the Mimic Technologies da Vinci Trainer) are available. In our training model, trainees must obtain 90% scores on the simulator exercises before proceeding on with the suturing training [5].

#### Advanced suturing training using biotissue

The advantages of suturing training using biotissue have been demonstrated to obtain proficiency in reconstructions of RPD [8, 9]. Advanced suturing training can improve errors and technical performance, leading to a shorter learning curve. In our training model, the trainees simulate anastomoses, such as PJ and HJ, and enhance suturing skills using the biotissue developed by the Ethicon and TMC Inc. (Osaka, Japan). Biotissue has been determined to be convenient, reproducible, and ubiquitous.

**Fig. 2:**
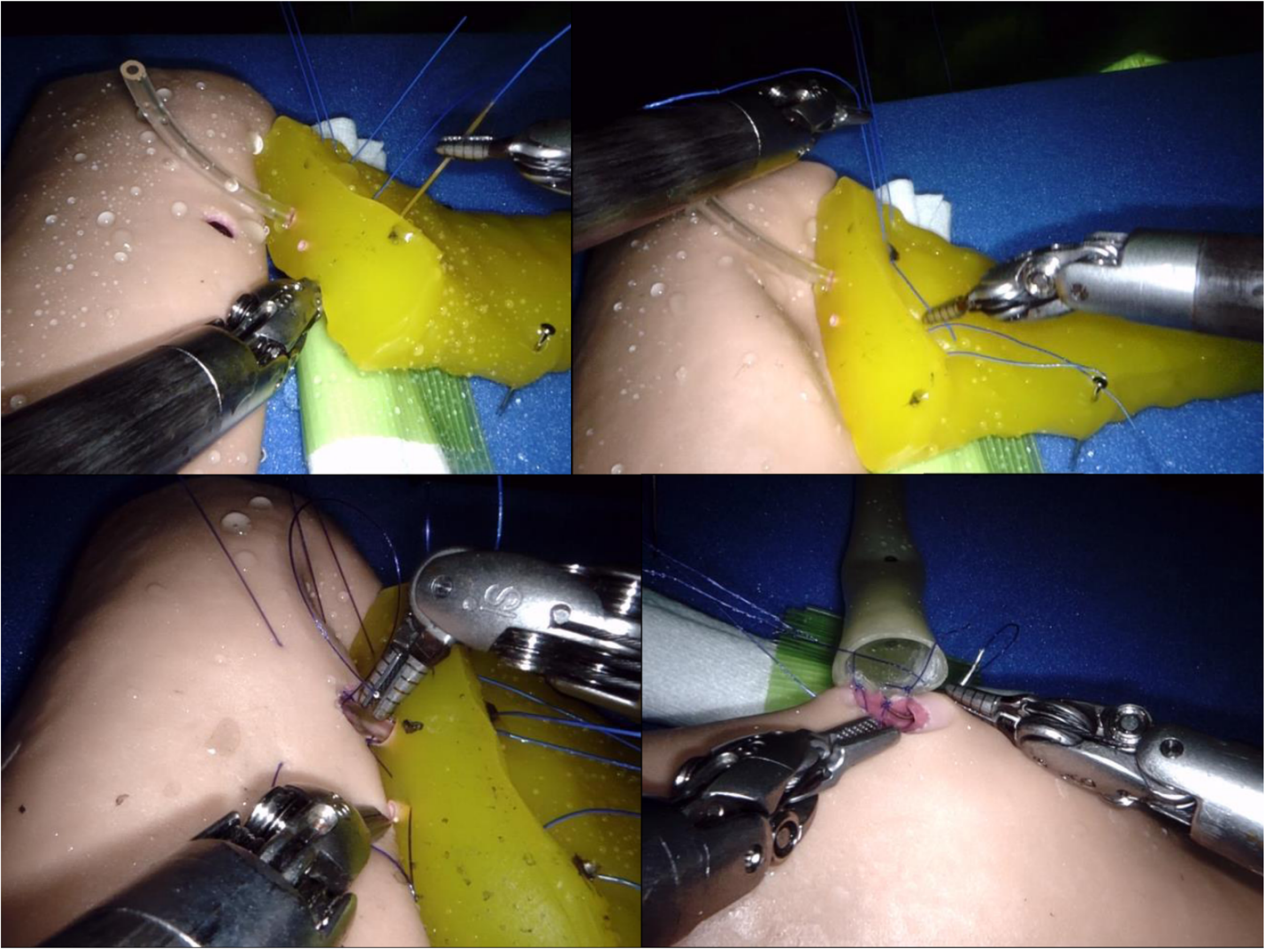
Biotissue curriculum for pancreaticojejunostomy and hepaticojejunostomy. The Pancreas Suture Model (TMC Inc., Osaka, Japan) is convenient, reproducible, and ubiquitous for suturing training of pancreaticojejunostomy

Our biotissue curriculum using the da Vinci Xi robotic system is shown in Fig. 2. Biotissue training is practical for anastomoses of PJ and HJ. The trainees can conduct more realistic training than simulation training and gain experience in handling the robotic system through biotissue training.

#### Surgical video review

The resection phase of RPD can be trained by observing various videos, including resections for several diseases, as well as troubleshooting. In addition, the reconstruction phase of the RPD can be standardized by watching several videos. The video library provided by the Pittsburgh group should help with the preparations prior to the operating room [10]. Furthermore, educational videos have been created for trainees, as shown in Supporting Video 1. Moreover, the video conferences among the multidisciplinary team would help to teach a sequence of the procedure.


Additional file 1: **Supporting Video 1**. Surgical technique of robotic pancreatoduodenectomy. Briefly our techniques can be divided into seven steps: Step 1, extended Kocher’s maneuver; Step 2, dissection of the hepatoduodenal ligament; Step 3, division of the pancreatic neck; Step 4, the uncinate dissection; Step 5, pancreaticojejunostomy; Step 6, hepaticojejunostomy; and Step 7, gastrojejunostomy.

### Standardization of surgical technique

**Fig. 3:**
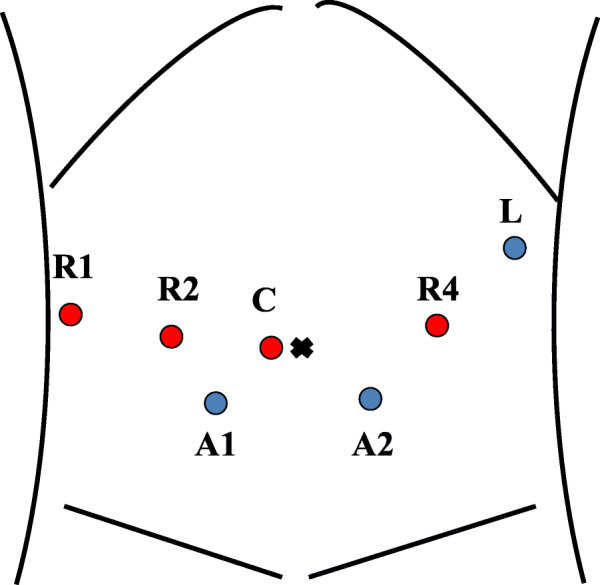
Trocar placement using the da Vinci Xi robotic system. R1, first robotic arm; R2, second robotic arm; R3, third robotic arm; C, camera; A1, trocar (5 mm) for an assistant; A2, trocar (12 mm) for an assistant; L, liver retractor

Our surgical techniques have been standardized based on experiences provided by the Erasmus MC, the Netherlands, and the University of Pittsburgh Medical Center, USA. Briefly, our RPD techniques can be divided into seven steps (Supporting Video 1). The patient is positioned in the supine position with the patient-side surgeon between the legs. After four robotic trocars at the umbilical level and two trocars (5 mm and 12 mm) for an assistant are inserted, the robot will be docked (Fig. 3).

#### Step 1: extended Kocher’s maneuver

The stomach is lifted cranially, and the omentum is opened extensively. After the mobilization of the right colon and kocherization of the duodenum, the inferior vena cava and left renal vein are exposed. Once the Treitz ligament is divided, the jejunum is pulled into the right upper quadrant space and transected with a linear stapler. Additionally, the stomach is divided for the subtotal stomach-preserving technique.

#### Step 2: dissection of the hepatoduodenal ligament

The hepatoduodenal ligament is dissected to obtain proper skeletonization of the vascular anatomy and to staple the gastroduodenal artery and bile duct. The gallbladder can be taken down at this step or later on. Hilar lymphadenectomy is performed in patients with malignant diseases.

#### Step 3: division of the pancreatic neck

The superior and inferior border of the pancreas on the superior mesenteric vein (SMV) are gently exposed to transect the pancreatic neck. The pancreas is divided using the monopolar curved scissors, followed by hemostasis from the pancreatic stump. Intraoperative pathological examination of the resection margin of the pancreatic duct can be performed.

#### Step 4: the uncinate dissection

The retraction of the pancreatic head is gently applied in order to detach the uncinate process. The nerve plexus around the superior mesenteric artery is dissected caudally to the cranial direction while dividing the inferior pancreaticoduodenal artery and several branches from the SMV. Finally, the specimen is extracted through the Pfannenstiel incision at this step.

#### Step 5: Pancreaticojejunostomy

**Fig. 4:**
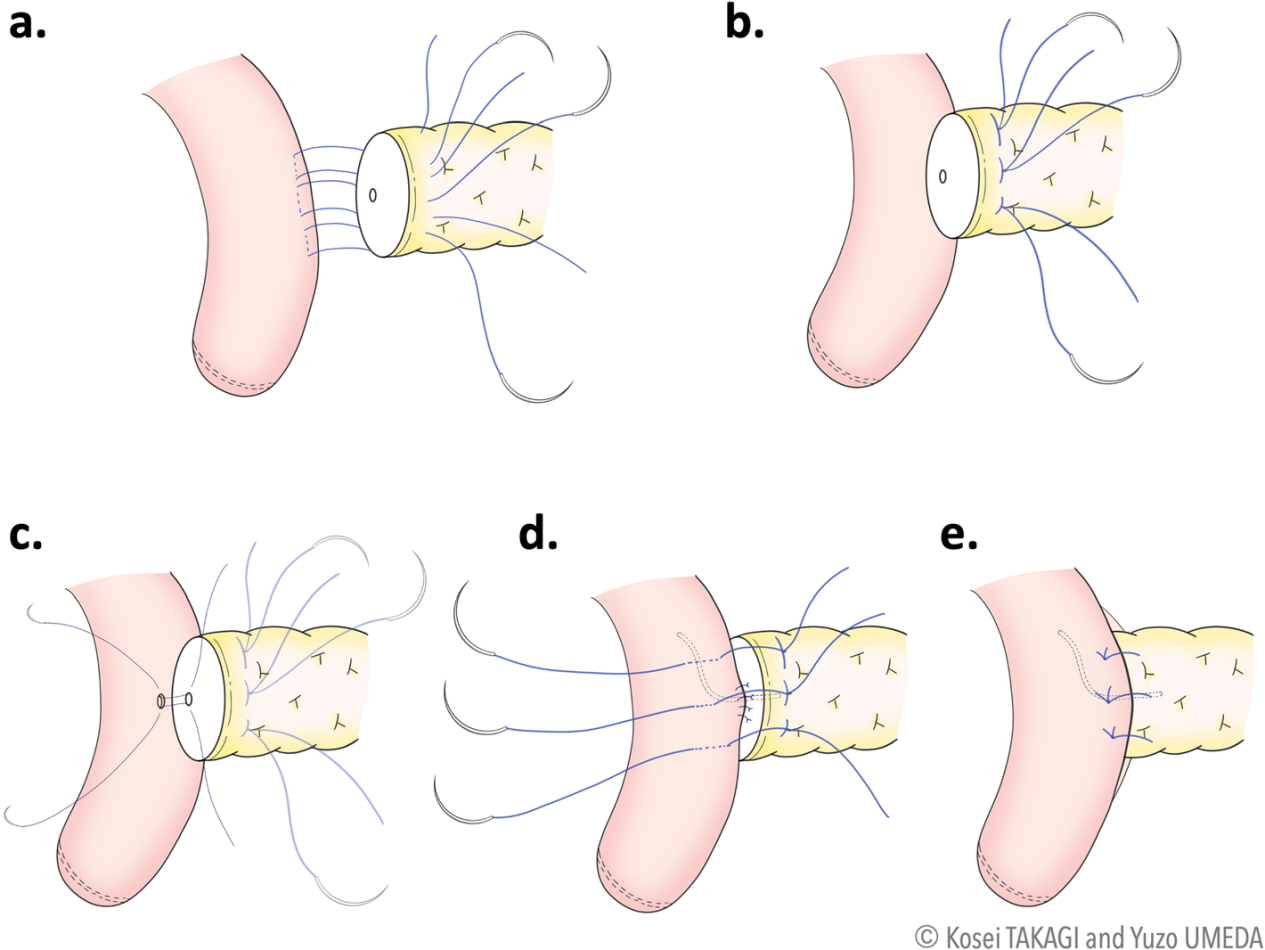
Schemes of robotic pancreaticojejunostomy with the modified Blumgart method. **a** Three U-sutures are established with 3-0 polypropylene sutures that are pierced through the pancreatic stump from front to back and reverted from back to front after the jejunal serosa was taken. **b** These U-sutures are tied gently to the pancreas. **c** The pancreatic duct-to-jejunum mucosa anastomosis was fashioned with interrupted 5-0 PDS sutures. A lost stent can be inserted into the main pancreatic duct. **d** The anterior wall of the jejunum is bitten with U-sutures. **e** The pancreatic stump is completely covered

The reconstruction commences with the PJ anastomosis (interrupted two-layer modified Blumgart method). After the mobilization of the pancreas, three U-sutures were established with 3-0 polypropylene sutures, pierced through the pancreatic stump from the front to the back and reverted from the back to the front after the jejunal serosa is taken (Fig. 4a). These U-sutures were tied gently on the pancreas (Fig. 4b). Subsequently, the pancreatic duct-to-jejunum mucosa anastomosis is fashioned with interrupted 5-0 PDS sutures (Fig. 4c). A lost stent of size 4.0 or 6.0 French can be inserted in the main pancreatic duct. The anterior wall of the jejunum is bitten with U-sutures and used to cover the pancreatic stump (Fig. 4d, e). The PJ schemes with the modified Blumgart method are depicted in Fig. 4.

#### Step 6: Hepaticojejunostomy

The HJ anastomosis is performed with interrupted sutures using 5-0 PDS. After the completion of the posterior layer, the lost stent can be placed in the anastomosis, following the anastomosis of the anterior layer.

#### Step 7: Gastrojejunostomy

**Fig. 5:**
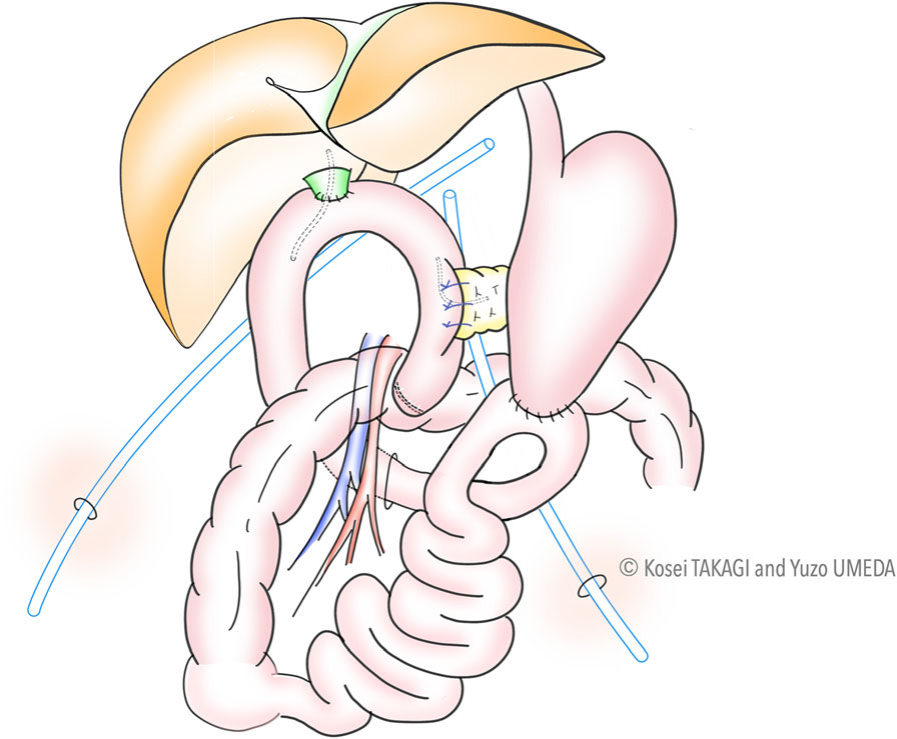
Overview of robotic pancreatoduodenectomy

The antecolic gastrojejunostomy is carried out with the robotic-sewn anastomosis. The stump of the stomach is sutured to the side of the jejunum with continuous two-layer sutures using 3-0 V-loc [11]. The overview of RPD after reconstructions is demonstrated in Fig. 5.

#### Safe implementation

The structured training program and knowledge on surgical techniques, including tips, tricks, and pitfalls, enable safe implementation of RPD in selected patients in high-volume centers. In clinical practice, troubles during the procedure will be faced at some point; therefore, sufficient case observations can help to learn how to deal with troubleshooting. Moreover, we suggest that surgeons should gain enough experience with RPD as an assistant, and participate as an operator with a stepwise approach under proctoring procedures (Fig. 1). After the successful setup, the same multidisciplinary team should be involved in at least the first 10 procedures that could lead to expansion of the program [5]. Furthermore, investigation of outcomes during the learning curve should be essential to monitor the safety and quality of care.

## Results and discussion

The present study demonstrates our surgical training model and surgical techniques of RPD in accordance with the Dutch training system in collaboration with the University of Pittsburgh Medical Center. Without dedicated individual and team training as well as understanding surgical techniques and robotic systems, safe implementation of RPD would not be possible.

With respect to the surgical training of RPD, it is needless to say that skillful techniques and sufficient knowledge on pancreatic surgery are required; additionally, it is necessary to acquire robot-specific techniques and knowledge that are different from laparoscopic surgery. Therefore, the development of a robot-specific training model is more important. The majority of our training models are arranged based on the Dutch training system. However, our originalities include creating our own educational videos, conducting video conferences among the multidisciplinary team, development of our own biotissue curriculum, and the simple standardization of surgical techniques into seven steps.

As RPD requires advanced surgical techniques, the optimization of training should play an important role. A recent notable review proposed a pyramid demonstrating three training phases for the implementation of minimally invasive pancreatic surgery [12]. In the first phase, the surgeon should develop basic skills and procedure-specific skills using the following tools: simulation, biotissue drills, video libraries, live case observations, and training courses. During the second phase, the surgeon should learn simpler procedures through index procedures, fellowships, and proctoring programs to ensure patient safety during the first procedures. Finally, the third phase aims to perform procedures safely as a clinical practice while minimizing the learning curve. Patient selection, skill assessment, feedback, and mentoring are of high importance in optimizing this phase. Their suggested model should be of aid, especially to surgeons who are not experienced with RPD to train with a stepwise approach using the structured multimodality training system.

In the clinical setting, the stepwise approach from an assistant to an operator should also contribute to the expansion of the RPD program. However, providing a specific number of procedures to be an independent assistant and operator would be cumbersome because the learning curve may be affected by several factors, including previous experiences in pancreatic surgery and minimally invasive surgery. The learning curve for RPD has been reported to be different from approximately 30 to 80 cases [13–16]. Therefore, at least 30 cases may be required to become an independent assistant or operator.

The Miami evidence-based guidelines encourage participation in a structured training program including virtual reality simulation, biotissue training to practice dissection and anastomoses, surgical video review, and on-site proctoring [1]. Furthermore, a recent interesting review suggested the importance of the structured multimodality training system for the safe implementation of minimally invasive pancreatic surgery [12]. Therefore, a multicenter structured training system should be established for surgeons and centers to introduce RPD in Japan.

## Conclusions

We developed a surgical training model of RPD based on experiences during the fellowship in the Netherlands. Our developed training model includes basic robotic training, simulation training, biotissue training, surgical video review, and clinical practice of RPD. Furthermore, the standardized surgical technique should help trainees to understand the tips, tricks, and pitfalls, leading to a shortened learning curve and safe implementation of RPD. Trainees will follow the program before performing procedures successfully and safely. Lastly, the nationwide structured training system should be developed to implement the program successfully and safely.

## Data Availability

All data generated or analyzed during this study are included in this published article.

## Abbreviations

PD: Pancreatoduodenectomy
RPD: Robotic pancreatoduodenectomy
HPB: Hepato-pancreato-biliary
PJ: Pancreaticojejunostomy
HJ: Hepaticojejunostomy
SMV: Superior mesenteric vein
